# A novel series of human dihydroorotate dehydrogenase inhibitors discovered by *in vitro* screening: inhibition activity and crystallographic binding mode

**DOI:** 10.1002/2211-5463.12658

**Published:** 2019-05-29

**Authors:** Ting Zeng, Zeping Zuo, Youfu Luo, Yinglan Zhao, Yamei Yu, Qiang Chen

**Affiliations:** ^1^ Cancer Center West China Hospital Sichuan University and Collaborative Innovation Center of Biotherapy Chengdu China

**Keywords:** crystal structure, dihydroorotate dehydrogenase, drug, inhibitor, pyrimidine biosynthesis

## Abstract

Human dihydroorotate dehydrogenase (DHODH), the enzyme that catalyzes the rate‐limiting step in *de novo* pyrimidine biosynthesis, is considered to be an attractive target for potential treatment of autoimmune disease and cancer. Here, we present a novel class of human DHODH inhibitors with high inhibitory potency. The high‐resolution crystal structures of human DHODH complexed with various agents reveal the details of their interactions. Comparisons with the binding modes of teriflunomide and brequinar provide insights that may facilitate the development of new inhibitors targeting human DHODH.

**Abbreviations**
C11DAO: *N*,*N*‐dimethylundecylamine *N*‐oxide
DDAO: *N*,*N*‐dimethyldecylamine *N*‐oxide
DHODH: dihydroorotate dehydrogenase
DHO: dihydroorotic acid
FMN: flavin mononucleotide

The dihydroorotate dehydrogenase (DHODH) is a key enzyme involved in the *de novo* pyrimidine biosynthetic pathway, which is conserved among all living organisms 1. DHODH uses flavin mononucleotide (FMN) as its redox cofactor and catalyzes the fourth step in this biosynthesis pathway, the conversion of l‐dihydroorotate to orotate. DHODHs have been shown to substantially be diverse in their structural, kinetic, and functional properties 1. Via phylogenetic analysis, DHODH could be clustered into two major classes: class 1 and class 2 1. The different classes differ also in their cell location: DHODHs from class 1 are found in the cytosol, whereas those from class 2 are membrane‐associated. Human DHODH belongs to class 2 and is anchored at the inner mitochondrial leaflet 2.

Inhibition of pyrimidine metabolism by selectively targeting DHODHs has been exploited in the development of new therapies against cancer, immunological disorders, bacterial and viral infections, and parasitic diseases 3, 4. Although a variety of inhibitors targeting human DHODH has been studied over the years, only leflunomide and its *in vivo* metabolite teriflunomide have been approved as human DHODH‐targeting drugs 4, 5, 6. The severe side effects, narrow therapeutic window, and inconsistent pharmacokinetics of the available DHODH inhibitors raise the need of new and more efficient human DHODH inhibitors 7, 8.

Here, we present a novel class of human DHODH inhibitors, which are based on 6‐isopropyl‐1,5,6,7‐tetrahydro‐4H‐benzo[d][1,2,3]triazol‐4‐one scaffold. *In vitro* inhibitory assay revealed that the compounds **1289** and **1291** have high potency against human DHODH. High‐resolution crystal structures of human DHODH and inhibitor complex elucidated their interactions. Our studies provide a solid structural basis for the design and development of new chemo‐diverse inhibitors targeting human DHODH.

## Materials and methods

### Chemical synthesis of compounds

#### 6‐Isopropyl‐1‐(2,2′,6‐trifluoro‐5′‐(hydroxymethyl)‐[1,1′‐biphenyl]‐4‐yl)‐1,5,6,7‐tetrahydro‐4H‐benzo[d][1,2,3]triazol‐4‐one (1289)

Under N_2_ atmosphere, a mixture of 4‐bromo‐3,5‐difluoroaniline (0.720 g, 3.49 mmol), (2‐fluoro‐5‐(hydroxymethyl)phenyl) boronic acid (889 mg, 5.23 mmol), Pd (dppf) Cl2.DCM (120 mg, 0.147 mmol), and potassium carbonate (1.45 g, 10.46 mmol) was dissolved in 15 mL of dioxane and 5 mL of H_2_O. The mixture was heated to 90 °C for overnight and then was allowed to cool to room temperature. The reaction was quenched with water and extracted with dichloromethane (3 × 100 mL). The combined organic layer was washed with brine, and then evaporated and concentrated *in vacuo*. The crude product was purified using silica gel chromatography with a petroleum ether/dichloromethane gradient to afford the desired product as a yellow oily liquid. (410 mg, 42.9% yield). ^1^H NMR (400 MHz, CDCl_3_) δ 7.52–7.47 (m, 1H), 7.44 (d, *J *=* *6.2 Hz, 1H), 7.33 (s, 2H), 7.22 (d, *J *=* *8.8 Hz, 1H), 4.76 (s, 2H), 3.66 (s, 2H). To a suspension of BF_3_·OEt_2_ (0.9 mL, 7 mmol) at −15 °C was added a solution of (4′‐amino‐2′,6,6′‐trifluoro‐[1,1′‐biphenyl]‐3‐yl) methanol (680 mg, 2.67 mmol) in THF (10 mL). After stirring for 30 min, tert‐BuONO (0.8 mL, 7 mmol) was added. After stirring the mixture at −15 °C for 1 h, the temperature was raised to 5 °C. Pentane (10 mL) was added, and the precipitate was collected by filtration and washed with pentane. The yellow solid was dissolved in MeCN (10 mL) and added to a solution of NaN_3_ (110 mg, 1.7 mmol) in water (2 mL) at room temperature. The mixture was stirred at 75 °C overnight and then allowed to cool to room temperature. The reaction mixture was quenched with water and extracted with dichloromethane (2 × 50 mL). The organic layer was dried with sodium sulfate and concentrated *in vacuo*. The crude product was purified using silica gel chromatography with a petroleum ether/dichloromethane gradient to afford the desired product as white solid (378 mg, 70.1% yield). ^1^H NMR (400 MHz, CDCl_3_) δ 7.52–7.47 (m, 1H), 7.44 (d, *J *=* *6.2 Hz, 1H), 7.33 (s, 2H), 7.22 (d, *J *=* *8.8 Hz, 1H), 4.76 (s, 2H). A mixture of 5‐isopropylcyclohexane‐1,3‐dione (106 mg, 0.69 mmol), (4′‐azido‐2′,6,6′‐trifluoro‐[1,1′‐biphenyl]‐3‐yl)methanol (192 mg, 0.69 mmol), and DBU (15 mol %) in CH_3_CN (10 mL) was stirred at 80 °C for 7–8 h. The reaction was quenched with water and extracted with dichloromethane (3 × 50 mL). The combined organic layer was washed with brine and then evaporated *in vacuo*. The crude product was purified using silica gel chromatography with a petroleum ether/dichloromethane gradient to afford the desired product as white powder (96 mg, 34.5% yield). ^1^H NMR (400 MHz, CDCl_3_) δ 7.52–7.47 (m, 1H), 7.44 (d, *J *=* *6.2 Hz, 1H), 7.33 (s, 2H), 7.22 (d, *J *=* *8.8 Hz, 1H), 4.76 (s, 2H), 3.10 (dd, *J *=* *12.0, 4.4 Hz, 1H), 2.89 (dd, *J *=* *10.8, 5.6 Hz, 1H), 2.76 (dd, *J *=* *13.6, 2.8 Hz, 1H), 2.49 (dd, *J *=* *13.2, 3.2 Hz, 1H), 2.22 (m, 1H), 1.83 (dd, *J *=* *6.4, 6.4 Hz, 2H), 1.04 (d, *J *=* *6.4 Hz, 6H); ^13^C NMR (101 MHz, CDCl_3_) δ 189.75,160.59, 144.26, 142.87, 137.08 (d, *J *=* *3.03 Hz), 136.49, 130.46, 129.99 (d, *J *=* *8.08 Hz), 116.23 (d, *J *=* *22. 22 Hz), 115.47 (d, *J *=* *20. 2 Hz), 107.15, 64.27, 58.48, 50.87, 42.65, 42.09, 31.74, 29.70, 25.34, 19.65 (d, *J *=* *8.08 Hz), 18.43.

#### 1‐(3′‐(Dimethylamino)‐2,6‐difluoro‐[1,1′‐biphenyl]‐4‐yl)‐6‐isopropyl‐1,5,6,7‐tetrahydro‐4H‐benzo[d][1,2,3]triazol‐4‐one (1291)

Compound **1291** was synthesized by a similar procedure as **1289**. ^1^H NMR (400 MHz, CDCl_3_) δ 7.36 (t, *J *=* *8.0 Hz, 1H), 7.29 (d, *J *=* *8.0 Hz, 2H), 6.83 (dd, *J *=* *12.0, 6.0 Hz, 3H), 3.08 (dd, *J *=* *16.6, 4.0 Hz, 1H), 3.00 (s, 6H), 2.91 (s, 1H), 2.76 (dd, *J *=* *13.2, 3.2 Hz, 1H), 2.50 (s, 1H), 2.27–2.18 (m, 1H), 1.83 (dd, *J *=* *13.0, 6.6 Hz, 1H), 1.03 (d, *J *=* *6.4 Hz, 6H). ^13^C NMR (101 MHz, CDCl_3_) δ 189.81, 161.76 (d, *J *=* *8.08 Hz), 161.68 (d, *J *=* *9.09 Hz), 159.27, 159.18, 150.54, 144.29, 142.76, 135.13, 129.19, 128.13, 120.98, 118.12, 113.98, 113.09, 107.36 (d, *J *=* *31.31 Hz), 107.26 (d, *J *=* *11.11 Hz), 53.43, 42.64, 42.11, 40.52, 31.74, 25.27, 19.64 (d, *J *=* *6.06 Hz).

### *In vitro* inhibitory activity of compounds

Human DHODH inhibition profiles are obtained from human DHODH‐inhibitor profiler services provided by ChemPartner (Shanghai, China).

### Protein preparation

Human DHODH was cloned into a vector derived from pET‐28a (+) (Novagen, Madison, WI, USA), which contains an N‐terminal SUMO tag and an N‐terminal His6 tag, and overexpressed in *Escherichia coli* strain Rosetta (DE3) (Novagen) at 18 °C for 18 h. The cells were harvested by centrifugation, and the cell pellet was resuspended in binding buffer (50 mm Tris/HCl pH 7.5, 500 mm NaCl, 0.33% Thesit, 10% glycerol, 1 mm PMSF). The cells were lysed by an ultrahigh‐pressure homogenizer (JNBIO) and centrifuged. The resultant supernatant was collected and loaded onto a Ni‐NTA column pre‐equilibrated with binding buffer. After washed with binding buffer supplemented with 20 mm imidazole to remove nonspecifically binding proteins, the target protein was eluted using binding buffer supplemented with 250 mm imidazole. The eluted target protein was collected and dialyzed against binding buffer with ULP1 protease (1 : 100) for 16 h at 8 °C to remove the SUMO tag. The digested protein was then passed through a Ni‐NTA column (GE Healthcare, Marlborough, MA, USA) to remove free SUMO tag, uncleaved protein, and ULP1 protease. The flow‐through was collected and was further purified via gel filtration (Superdex 200 10/300 GL; GE Healthcare) in a buffer consisting of 50 mm HEPES, pH 7.5, 400 mm NaCl, 10% glycerol, 1 mm EDTA, and 0.05% Thesit, on an AKTA system (GE Healthcare). The purified proteins were concentrated to 20 mg·mL^−1^ and stored at −80 °C until use.

### Cocrystallization of human DHODH and inhibitors

Purified DHODH was incubated with 2 mm dihydroorotic acid (DHO), 40 mm *N*,*N*‐dimethylundecylamine N‐oxide (C11DAO), 20.8 mm *N*,*N*‐dimethyldecylamine *N*‐oxide (DDAO), and fivefold small molecule inhibitor for 2 h at 4 °C. Crystals of DHODH and inhibitor were grown using the hanging‐drop vapor diffusion method at 20 °C, in a buffer consisting of 0.1 m acetate pH 4.6, 1.8–2.0 m ammonium sulfate, and 30–35% glycerol. Cubic crystals appeared after a week.

### Data collection and structure determination

Crystals were fished directly from the growing drop and flash‐frozen in liquid nitrogen. Diffraction data were collected on beamline BL19U1 of National Facility for Protein Science Shanghai (NFPS) at Shanghai Synchrotron Radiation Facility. The data collected were processed by the hkl‐3000 program suite 9. Details of the data collection and processing statistics are summarized in Table 1. Structures were determined by molecular replacement using the human DHODH structure (PDB ID 1D3G) as search model. Structure refinement and model building were performed with phenix 10 and coot 11. All models were validated with molprobity 12. All structure figures were prepared with pymol (https://pymol.org ).

**Table 1:** Chemical structures and *in vitro* inhibitory activities of human DHODH inhibitors.

Compound	Chemical formula	IC_50_ (nm)
Brequinar	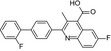	5.2
Teriflunomide	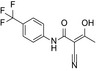	388^a^
**1289**	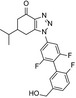	171
**1291**	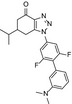	39

^a^IC_50_ value of teriflunomide is from the reference 17.

## Results and Discussion

### A novel class of potent inhibitors of human DHODH

Biphenyl moiety was observed in several DHODH inhibitors, such as leflunomide, brequinar, and vidofludimus 13, 14, 15, and plays a key role in ligand–DHODH interactions. Therefore, the compounds with a biphenyl moiety in our in‐house library were screened for their DHODH inhibitory effects. Finally, the screens led to the discovery of a series of compounds which have a 6‐isopropyl‐1,5,6,7‐tetrahydro‐4H‐benzo[d][1,2,3]triazol‐4‐one scaffold, which are significantly different from available DHODH inhibitors.

**Scheme 1:**
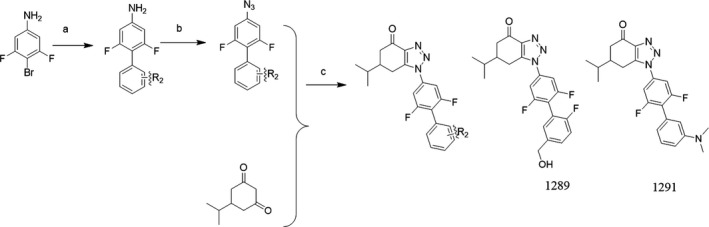
Synthesis of compounds **1289** and **1291**. Reagents and conditions: (a) aryl boronic acid or borate, Pd(dppf)Cl_2_, DCM, Cs_2_ CO_3_ or K_2_ CO_3_, 1,4‐dioxane/H_2_O, 100 °C, overnight; (b) (i) boron trifluoride diethyl etherate, tert‐butyl nitrite, THF, −20 °C to rt, 2 h; (ii) NaN_3_, CH_3_ CN/H_2_O; and (c) 5‐isopropylcyclohexane‐1,3‐dione, DBU, CH_3_ CN, 80 °C, 3–4 h.

Recently, a series of hDHODH inhibitors were discovered by scaffold‐hopping strategy or structural modification based on previous reported lead compounds 16, 17 In our instance, the active compounds were selected based on *in vitro* screening. Our screening discovered a novel class of human DHODH inhibitors, which have a 6‐isopropyl‐1,5,6,7‐tetrahydro‐4H‐benzo[d][1,2,3]triazol‐4‐one scaffold. Among these compounds, **1289** and **1291** have shown high *in vitro* inhibitory activity against human DHODH with nm range IC_50_ values (Table 1). The IC_50_ values of other two compounds, the approved and market‐established drug teriflunomide and one of the strongest known DHODH inhibitors brequinar, are presented here for comparison. The synthesis of **1289** and **1291** has been shown in Scheme 1.

### Crystal structures of DHODH‐inhibitor complex

**Figure 1:**
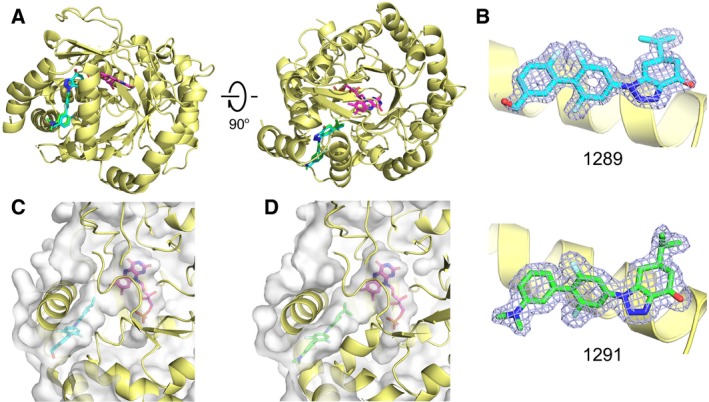
Crystal structures of human DHODH in complex with inhibitors. (A) Overall structures of human DHODH complexed with compound **1289** or **1291**. Compounds **1289** (cyan) and **1291** (green) are shown as sticks and superimposed. FMN is shown as sticks and colored in purple. (B) The electron densities of inhibitors **1289** and **1291**. The Fo‐Fc omit map is colored light blue and contoured at 3 σ. The compounds **1289** (C) and **1291** (D) are located at a tunnel connecting to the FMN cavity. The surface of DHODH is colored in semitransparent white to show the long tunnel occupied by the inhibitors and FMN.

To elucidate the binding modes of these two inhibitors, we determined the crystal structures of human DHODH complexed with **1289** or **1291** to 1.6 and 1.85 Å resolutions, respectively (Fig. 1A). Details of the data collection and refinement statistics are summarized in Table 2. The high‐resolution and clear density maps enable us to determine the position and orientation of these inhibitors unambiguously (Fig. 1B). Superposition of these two structures shows an identical binding mode for the two inhibitors (Fig. 1A).

**Table 2:** Crystallography data collection and refinement statistics.

	DHODH‐1289	DHODH‐1291
Data collection		
Space group	P 32 2 1	P 32 2 1
Cell dimensions		
*a*,* b*,* c* (Å)	90.8, 90.8, 122.8	90.6, 90.6, 122.8
α, β, γ (°)	90.0, 90.0, 120.0	90.0, 90.0, 120.0
Resolution (Å)	50.0–1.60 (1.63–1.60)^a^	50.0–1.85 (1.89–1.85)
*R*_meas_	0.176 (0.768)	0.132 (0.795)
*I*/σ*I*	13.7 (2.5)	13.0 (2.0)
Completeness (%)	100.0 (100.0)	100.0 (100.0)
Redundancy	10.0 (9.5)	9.9 (9.6)
Refinement		
Resolution (Å)	48.4–1.60	41.0–1.85
No. of reflections	77, 563	49,917
*R*_work_/*R*_free_	0.157/0.176	0.168/0.195
No. of atoms		
Protein	2,768	2,821
Ligand/ion	155	112
Water	330	282
R.m.s deviations		
Bond lengths (Å)	0.007	0.007
Bond angles (°)	1.087	1.018

^a^Highest resolution shell is shown in parentheses.

**Figure 2:**
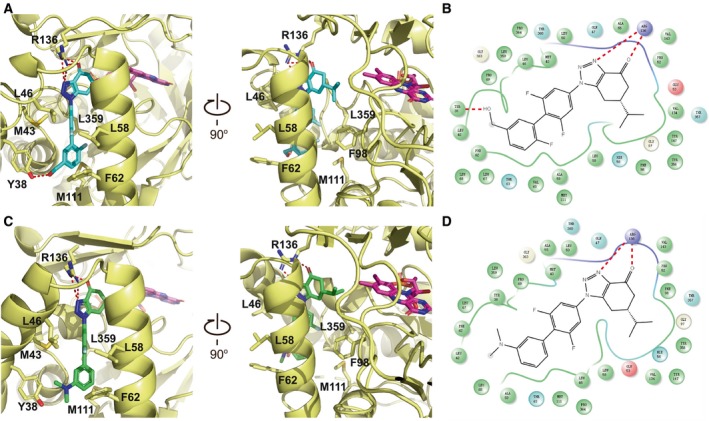
The binding environment of human DHODH inhibitors. (A, C) Interactions between human DHODH and the inhibitors. The compounds **1289** (cyan), **1291** (green) and FMN (purple) are shown as sticks. Key residues that make interactions with the inhibitors are shown as sticks and labeled. The hydrogen bonds are shown as red dashed lines. (B, D) Schematic plots of the interactions of compounds **1289** and **1291** with human DHODH. Red dashed lines show hydrogen bonding interactions. The plot was prepared with Schrödinger Suites.

Human DHODH has an α/β‐barrel fold (Fig. 1A), and the inhibitors and FMN are located at a tunnel within DHODH (Fig. 1C,D). Our structures reveal the detailed interactions between the inhibitors and human DHODH (Fig. 2). Both compounds are stabilized by a substantial number of hydrophobic interactions involving M43, L46, L58, F62, F98, M111, and L359. Compound **1289** forms hydrogen bonds with the side chains of R136 and Y38 (Fig. 2A). Compound **1291** forms hydrogen bonds with R136 as same as compound **1289**, but loses the hydrogen bond with Y38 due to the substitution of the hydroxymethyl group for the *N*,*N*‐dimethyl group (Fig. 2C). Meanwhile, such substitution reinforces the hydrophobic interactions between **1291** and the local hydrophobic environment, which may contribute to the lower IC_50_ value of this inhibitor (Table 1). The full binding environments of inhibitors **1289** and **1291** have been shown in Fig. 2B and D, respectively.

### Comparison of the binding modes of 1289, 1291, teriflunomide, and brequinar

**Figure 3:**
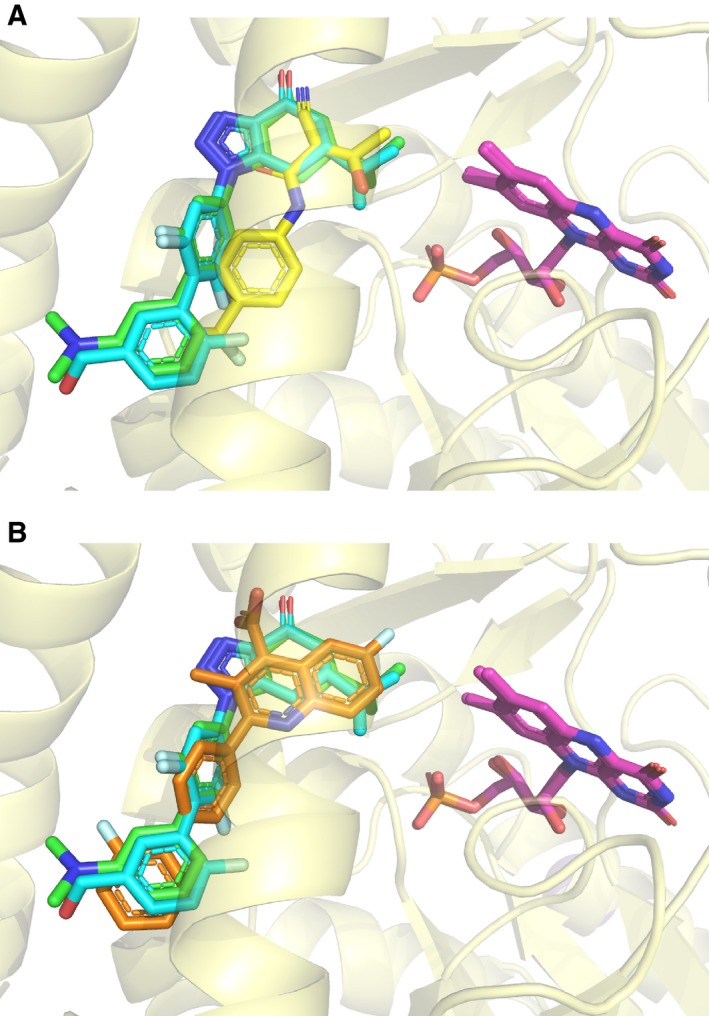
Comparison of the binding modes of inhibitors **1289** and **1291** with teriflunomide and brequinar. (A) Superposition of the complex structures of DHODH‐**1289**, DHODH‐**1291,** and DHODH–teriflunomide (PDB ID 1D3H). (B) Superposition of the complex structures of DHODH‐**1289**, DHODH‐**1291,** and DHODH–brequinar (PDB ID 1UUO). The compounds **1289**,** 1291,** teriflunomide, and brequinar are colored in cyan, green, yellow, and orange, respectively. FMN is colored in purple.

Comparison with the previously solved DHODH–teriflunomide and DHODH–brequinar complex structures has been carried out by superposition (Fig. 3). The FMN molecules from different structures are completely overlapped, suggesting the superposition is well performed. The two inhibitors we present here adopt a brequinar‐like binding mode, which is different from that of teriflunomide (Fig. 3). The biphenylic moiety of **1289** and **1291** occupied a very similar position as that of brequinar (Fig. 3B). However, the isopropyl group of **1289** and **1291** is mimicking the isopropyl alcohol group of teriflunomide, which is approaching to the FMN cofactor (Fig. 3A).

It has been shown that the DHODH‐inhibitor binding site has intrinsic plasticity 18, so human DHODH may accommodate a diverse range of inhibitors, rationalizing the strategy of designing inhibitors with diverse scaffolds. The high‐resolution structures of human DHODH‐inhibitor complex we report here elucidate the interactions between these new inhibitors and their target, and thus facilitate the design and development of novel, efficient, and chemo‐diverse inhibitors for human DHODH. Further *in vivo* efficacy studies and compound optimization are ongoing to evaluate this novel class of human DHODH inhibitors.

## Author contributions

QC and YY conceived and designed the project. TZ, ZZ, and YY acquired the data. YL, YZ, and QC analyzed and interpreted the data. QC wrote the paper.

## Conflict of interest

The authors declare no conflict of interest.

## References

[1] Reis RAG , Calil FA , Feliciano PR , Pinheiro MP and Nonato MC (2017) The dihydroorotate dehydrogenases: past and present. Arch Biochem Biophys 632, 175–191. 28666740 10.1016/j.abb.2017.06.019

[2] Jones ME (1980) Pyrimidine nucleotide biosynthesis in animals: genes, enzymes, and regulation of UMP biosynthesis. Annu Rev Biochem 49, 253–279. 6105839 10.1146/annurev.bi.49.070180.001345

[3] Vyas VK and Ghate M (2011) Recent developments in the medicinal chemistry and therapeutic potential of dihydroorotate dehydrogenase (DHODH) inhibitors. Mini Rev Med Chem 11, 1039–1055. 21861807 10.2174/138955711797247707

[4] Munier‐Lehmann H , Vidalain PO , Tangy F and Janin YL (2013) On dihydroorotate dehydrogenases and their inhibitors and uses. J Med Chem 56, 3148–3167. 23452331 10.1021/jm301848w

[5] Davis JP , Cain GA , Pitts WJ , Magolda RL and Copeland RA (1996) The immunosuppressive metabolite of leflunomide is a potent inhibitor of human dihydroorotate dehydrogenase. Biochemistry 35, 1270–1273. 8573583 10.1021/bi952168g

[6] Schattenkirchner M (2000) The use of leflunomide in the treatment of rheumatoid arthritis: an experimental and clinical review. Immunopharmacology 47, 291–298. 10878295 10.1016/s0162-3109(00)00194-6

[7] Lolli ML , Sainas S , Pippione AC , Giorgis M , Boschi D and Dosio F (2018) Use of human dihydroorotate dehydrogenase (hDHODH) inhibitors in autoimmune diseases and new perspectives in cancer therapy. Recent Pat Anticancer Drug Discov 13, 86–105. 29119937 10.2174/1574892812666171108124218

[8] Madak JT , Bankhead A III , Cuthbertson CR , Showalter HD and Neamati N (2019) Revisiting the role of dihydroorotate dehydrogenase as a therapeutic target for cancer. Pharmacol Ther 195, 111–131. 30347213 10.1016/j.pharmthera.2018.10.012

[9] Minor W , Cymborowski M , Otwinowski Z and Chruszcz M (2006) HKL‐3000: the integration of data reduction and structure solution–from diffraction images to an initial model in minutes. Acta Crystallogr D Biol Crystallogr 62, 859–866. 16855301 10.1107/S0907444906019949

[10] Adams PD , Afonine PV , Bunkóczi G , Chen VB , Davis IW , Echols N , Headd JJ , Hung LW , Kapral GJ , Grosse‐Kunstleve RW *et al* (2010) PHENIX: a comprehensive Python‐based system for macromolecular structure solution. Acta Crystallogr D Biol Crystallogr 66, 213–221. 20124702 10.1107/S0907444909052925 PMC2815670

[11] Emsley P and Cowtan K (2004) Coot: model‐building tools for molecular graphics. Acta Crystallogr D Biol Crystallogr 60, 2126–2132. 15572765 10.1107/S0907444904019158

[12] Chen VB , Arendall WB III , Headd JJ , Keedy DA , Immormino RM , Kapral GJ , Murray LW , Richardson JS and Richardson DC (2010) MolProbity: all‐atom structure validation for macromolecular crystallography. Acta Crystallogr D Biol Crystallogr 66, 12–21. 20057044 10.1107/S0907444909042073 PMC2803126

[13] Herrmann ML , Schleyerbach R and Kirschbaum BJ (2000) Leflunomide: an immunomodulatory drug for the treatment of rheumatoid arthritis and other autoimmune diseases. Immunopharmacology 47, 273–289. 10878294 10.1016/s0162-3109(00)00191-0

[14] Liu S , Neidhardt EA , Grossman TH , Ocain T and Clardy J (2000) Structures of human dihydroorotate dehydrogenase in complex with antiproliferative agents. Structure 8, 25–33. 10673429 10.1016/s0969-2126(00)00077-0

[15] Baumgartner R , Walloschek M , Kralik M , Gotschlich A , Tasler S , Mies J and Leban J (2006) Dual binding mode of a novel series of DHODH inhibitors. J Med Chem 49, 1239–1247. 16480261 10.1021/jm0506975

[16] Madak JT , Cuthbertson CR , Miyata Y , Tamura S , Petrunak EM1 , Stuckey JA , Han Y , He M , Sun D , Showalter HD *et al* (2018) Design, synthesis, and biological evaluation of 4‐quinoline carboxylic acids as inhibitors of dihydroorotate dehydrogenase. J Med Chem 61, 5162–5186. 29727569 10.1021/acs.jmedchem.7b01862 PMC8859982

[17] Sainas S , Pippione AC , Lupino E , Giorgis M , Circosta P , Gaidano V , Goyal P , Bonanni D , Rolando B , Cignetti A *et al* (2018) Targeting myeloid differentiation using potent 2‐hydroxypyrazolo[1,5‐ a]pyridine scaffold‐based human dihydroorotate dehydrogenase inhibitors. J Med Chem 61, 6034–6055. 29939742 10.1021/acs.jmedchem.8b00373

[18] Walse B , Dufe VT , Svensson B , Fritzson I , Dahlberg L , Khairoullina A , Wellmar U and Al‐Karadaghi S (2008) The structures of human dihydroorotate dehydrogenase with and without inhibitor reveal conformational flexibility in the inhibitor and substrate binding sites. Biochemistry 47, 8929–8936. 18672895 10.1021/bi8003318

